# Supplementary material for: Temperature vegetation dryness index (TVDI) for drought monitoring in the Guangdong Province from 2000 to 2019
Source: PeerJ. 2023 Dec 18;11:e16337. doi: 10.7717/peerj.16337 (PMC10734433; doi:10.7717/peerj.16337)
Supplement: Supplemental Information 2 [file peerj-11-16337-s002.doc]

**­Temperature Vegetation Dryness Index （TVDI）for Drought monitoring in the Guangdong Province from 2000 to 2019**

Ailin Chen^1,2,*^, JiaJun Jiang^3, *^, Yong Luo^1,2^, Guoqi Zhang^4^ ,Bin Hu^1,2^,Xiao Wang^5^,Shiqi Zhang^6,7^

^1^ Sichuan Earthquake Agency, Chengdu, China

^2^ Chengdu Institute of Tibetan Plateau Earthquake Research, China Earthquake Administration, Chengdu, China

^3^ Asia Pacific University of Technology & Innovation, Kuala Lumpur, Malaysia

^4^ School of Emergency Management, Xihua University, Chengdu, China

^5^ School of Architecture and Civil Engineering, Chengdu University, Chengdu, China

^6^ College of Earth Sciences, Chengdu University of Technology, Chengdu, China;

^7^ Department of Geosciences and Geography, University of Helsinki, Helsinki,Finland;

Corresponding Author:

Ailin Chen^1,2,*^

No. 29, Section 3, Renmin South Road, Chengdu, Sichuan, 610044, China

Email address: cal_gis@163.com

Ailin Chen and JiaJun Jiang contributed equally to this work.

Please do not submit this one-page document. Your cover page must be included in your main manuscript.

Copy and paste the above text into the first page of your manuscript and then replace the text with your author information.
